# The Safety of Acupuncture in Patients with Cancer Therapy–Related Thrombocytopenia

**DOI:** 10.1089/acu.2015.1099

**Published:** 2015-06-01

**Authors:** Paul A. Cybularz, Karen Brothers, Gurneet M. Singh, Jennifer L. Feingold, Mark E. Lewis, Michelle L. Niesley

**Affiliations:** Cancer Treatment Centers of America at Eastern Regional Medical Center, Philadelphia, PA.

**Keywords:** Thrombocytopenia, Oncology, Acupuncture, Platelets, Safety

## Abstract

**Background:** Acceptance of acupuncture as an efficacious integrative modality for oncology-related side-effect management is rapidly expanding. It is imperative that guidelines regarding safe treatment supported by clinical experience are established. Oncology patients frequently experience thrombocytopenia as a side-effect of chemotherapy or radiation. However, safety data for acupuncture in adult patients with cancer who are thrombocytopenic is lacking.

**Materials and Methods:** The medical records of 684 patients who received acupuncture treatments in an established acupuncture program at a private cancer treatment hospital were reviewed for adverse events occurring within the context of thrombocytopenia.

**Results:** Of 2135 visits eligible for evaluation, 98 individual acupuncture visits occurred in patients with platelet counts <100,000/μL, including nine visits in which platelet counts were <50,000/μL. No adverse events of increased bruising or bleeding were noted. Medications and nutritional supplements or botanicals that may influence coagulation were also tabulated, with no apparent adverse events in this patient population.

**Conclusions:** Discrepancies in the literature highlight the need to create cohesive safety guidelines backed by clinical research, specifically for groups at higher risk for adverse events. The preliminary evidence put forth in this study lays the foundation that supports the notion that acupuncture can be used safely with a high-need oncology population within an integrated model of care. In this descriptive retrospective case series of adult oncology patients with thrombocytopenia, no adverse events of increased bruising or bleeding were documented. Prospective trials are needed to confirm these initial observations.

## Introduction

Acupuncture is a branch of Traditional Chinese Medicine (TCM) that involves the insertion of fine filiform needles into specific points in the body to elicit a desired physiologic response. While the active mechanism(s) of acupuncture have yet to be defined fully, studies suggest that many of acupuncture's biomedical effects can be attributed to influences on the connective tissue, cytokine stimulation, adenosine modulation, neuromodulation, opioid production, and endorphin production.^1^

Despite acupuncture's growing acceptance and application since its endorsement by the National Institutes of Health in 1997,^2^ physicians often withhold referrals citing the desire for larger scale studies on safety and efficacy. Acupuncture has been shown to be safe and effective across a variety of international clinical settings. A cumulative review summarized the results of 12 studies which examined >1 million acupuncture treatments, and concluded that the risk of a serious adverse reaction is ∼0.05 per every 10,000 treatments.^3^ Side-effects most frequently associated with acupuncture treatments include—but are not limited to—hematoma, soreness at insertion site, localized skin irritation, or faintness. As patients with cancer have demonstrated a growing interest in utilizing acupuncture to manage oncology-related side-effects,^4^ a need exists to solidify clear evidence-based safety guidelines specific to this population.

Thrombocytopenia is commonly encountered in the oncology population. It is generally defined as a patient having <150,000 platelets per μL (normal range: 150,000/μL–415,000/μL). Thrombocytopenia in patients with cancer often results from either the myelosuppressive effects of radiation and chemotherapy, or infiltration of the patient's bone marrow by the malignancy.^5^ Acupuncture is commonly contraindicated in thrombocytopenic patients because of the concern regarding increased bleeding. Many academic institutions and community hospitals have identified 50,000/μL as the threshold at which acupuncture is contraindicated, based on the clinical practice guidelines of the American Society of Clinical Oncology.^6^ However, a 2010 retrospective review in pediatric oncology patients suggests that acupuncture may be safe at lower platelet counts.^7^ While caution in this situation is certainly warranted, appropriate guidelines based on a larger sample size will help clinicians identify patients confidently who could benefit safely from this emerging modality.

The largest study to date is a retrospective review that evaluated the safety of acupuncture in 32 children and adolescents with oncology-related thrombocytopenia.^5^ No serious adverse events were reported after a total of 237 acupuncture sessions, 112 of which occurred in patients with platelets <100,000/μL. Of note, 48 sessions occurred in patients with platelet counts <20,000/μL. The authors of the study noted that their results have been owing to the experience of the practitioners, the depth of penetration, the method of manipulation, or the type of needle used. The study justifies the need for a larger, more-conclusive investigation.

Because of the lack of comparative data in adult patients with cancer, the present study was conducted to establish the baseline assessment for the safe application of acupuncture in adult oncology patients with thrombocytopenia.

## Methods

After acquiring institutional review board approval, medical charts were reviewed from patients who received treatment with acupuncture at Cancer Treatment Centers of America at the Eastern Regional Medical Center (ERMC), during the period from March 2012 to August 2013. ERMC is a private cancer-treatment hospital with an established acupuncture program. Policies and procedures require a physician's order prior to initiating acupuncture therapy. All treatments were performed by 1 of 3 licensed acupuncturists at ERMC. Prior to treatment, the acupuncturist conducted a chart review and a thorough TCM intake, which included a combination of tongue and pulse diagnosis, channel palpation, chief-complaint assessment, and a review of systems. Acupuncture points, depths (generally no greater than 1 cun), applied needle manipulation, and retention time were chosen at the practitioner's discretion in accordance with TCM theory and ERMC guidelines. Acupuncture points were primarily located on the extremities, face, and scalp, with depths not exceeding >½″. Needles were retained for 15–30 minutes dependent upon patient needs. All patients were treated with Serin type #1, #2, and #3 disposable needles (0.16-mm, 0.18-mm, and 0.20-mm diameter) and 15-, 30-, or 50-mm length (SEIRIN-America, Weymouth, MA).

The details of TCM assessment and treatment were documented in the medical charts, including—but not limited to—chief complaint(s), TCM diagnosis (pulse and tongue assessment), acupuncture-point selection, and number of needles used per acupuncture treatment. The occurrence of acute adverse effects (e.g., hematoma, bleeding, pain, nausea, fainting, dizziness, vomiting, headache, anxiety, and localized skin irritation) during or immediately after needle removal were recorded by the acupuncturist at the time of treatment.

For each individual acupuncture treatment session, the patient chart was evaluated to determine if a platelet count was documented within 48 hours prior to or after the session. If data on platelet levels were available for visits in which patients had platelet levels <100,000/μL, the data collected for the purposes of this study included patient demographics (gender, age at time of treatment, and ethnicity), date of acupuncture visit(s), cancer type, date of oncology diagnosis, oncology treatment at time of acupuncture visit(s), location of acupuncture treatment (inpatient or outpatient), and platelet levels.

It is important to note that ERMC offers integrative medicine services in conjunction with standard oncology care. The supervised use of herbal and dietary supplements is incorporated into each patient's standard oncology treatment plan. While these additional supplements are prescribed to improve health outcomes, they are not pharmacologically inert and may affect a patient's risk for bleeding either directly or indirectly.^8^ Therefore, concurrent use of medications and/or nutraceuticals/botanicals that can affect clotting or platelet counts were also recorded to capture additional factors influencing the potential for adverse events (Table 1).

**Table 1.** Medications and Supplements That May Affect Clotting and/or Platelet Count

*Medications*	*Nutraceuticals & botanicals*	
Vicodin^®^	*Dong quai* (*Angelica sinensis*)	Grapefruit
Norco^®^	EDTA	Kava (*Piper methysticum*)
Darvocet^®^	Fish oil	Licorice (*Glycyrrhiza* spp.)
Percocet^®^	Cod liver oil	Milk thistle (*Silybum marianum*)
Oxycontin^®^	Flax (*Linum usitatissimum*) oil	Nattokinase
Ibuprofen (Advil^®^)	Evening primrose (*Oenothera biennis*) oil	Oregon grape (*Mahonia* spp.)
Aleve^®^	Borage (*Borago officinalis*) oil	*Pau d'Arco* (*Tabebuia impetignosa*)
Motrin^®^	Hemp (*Cannabis sativa*) oil	Quercetin
Plavix^®^	DHA	Red clover
Coumadin^®^	EPA	Resveratrol
Warfarin	Gamma linolenic acid	Serrapeptidase
Aspirin	Krill oil	St. John's wort (*Hypericum perforatum*)
Clopidogrel, Plavix^®^	Vitamin E	Turmeric (curcumin; *Circuma* spp.)
Heparin, Lovenox^®^	Vitamin K	DHEA
Prednisone	American ginseng (*Panax quinquefolius*)	Glucosamine products
Solumedrol^®^	Ginkgo (*Ginkgo biloba*)	Willow bark (*Salix alba*)
Dexamethasone	Garlic (*Camillia sinesis*)	Bromelain
Prednisolone	Ginger (*Zingiber officinale*)	Capsicum (cayenne; *Capsicum* spp.)
	Goldenseal (*Hydrastis canadensis*)	Cascara

EDTA, ethylenediaminetetraacetic acid; DHA, docosahexaenoic acid; EPA; eicosapentaenoic acid; DHEA, dehydroepiandrosterone.

## Results

**FIG. 1.**
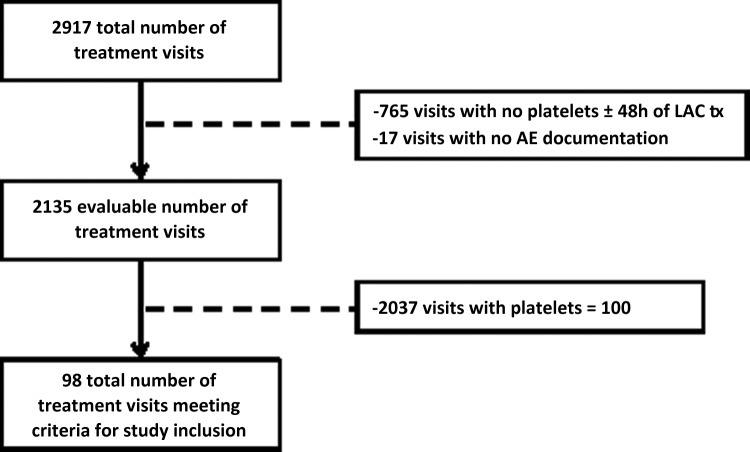
Inclusion and exclusion criteria for study. Tx, treatment.

A total of 2917 acupuncture visits for 684 unique patients occurred from March 2012 to August 2013. Data from 782 visits were not evaluable either because there were no data on platelet count available within 48 hours pre- or post-acupuncture treatment or, in 17 cases, for which the adverse-event section of the charts had not been completed (Fig. 1). The resulting 2135 treatment visits were then evaluated to determine if each patient had a documented platelet level of <100,000/μL within 48 hours of the visit. A total of 98 sessions were subsequently identified as meeting the inclusion criteria, as 2037 sessions occurred in patients with platelet levels >100,000/μL.

Demographics for individuals receiving acupuncture with thrombocytopenia versus the entire patient population are outlined in Table 2. The median number of acupuncture sessions per patient was 1 (range: 1–8). Ninety-eight sessions involved 69 patients who had platelet levels <100,000/μL within 48 hours of the visit. Based on the prior work in pediatric oncology by Ladas et al.,^7^ the same thrombocytopenia scale was implemented. Platelets of ≤20,000/μL were designated as “severe” thrombocytopenia. “Moderate” thrombocytopenia was defined as platelet levels of 21,000–50,000/μL, while platelet levels of 51,000–100,000/μL constituted “mild” thrombocytopenia. Table 3 shows the distribution of acupuncture treatments that involved patients with and without thrombocytopenia. The median number of needles used per acupuncture session in patients with moderate and mild thrombocytopenia were 10 (5–14) and 10 (2–35), respectively.

**Table 2.** Patient Demographics

*Visits*	*All patients*	*Patients with thrombocytopenias*
**Total visits**	2918	98
**Unique patients**	684	69
**Age (years)**		
Median Age	55	57
Range	20–86	25–70
**Gender**		
Male	230	27
Female	454	42
**Race/ethnicity**		
Caucasian		53 (76.8%)
Black, non-Hispanic		10 (14.5%)
Hispanic		1 (1.5%)
Asian		1 (1.5%)
Other/unknown		4 (5.8%)
**Cancer types**		
Breast		14 (20.6%)
Gynecologic		11 (16.2%)
Ovarian		4 (5.9%)
Uterine		5 (7.4%)
Vulvar		1 (1.5%)
Lung		10 (14.7%)
Colorectal		9 (13.2%)
Pancreatic		6 (8.8%)
Other		18 (26.5%)
**Location**		
Inpatient		28 (28.6%)
Outpatient		70 (71.4%)

**Table 3.** Distribution of Acupuncture Treatments During Severe, Moderate, and Mild Thrombocytopenia

*Thrombocytopenia grade*	*# of treatments*	*Median # of needles (range)*	*Mean platelets/μL (range)*
Severe (<20,000/μL)	0	0	0
Moderate (21,000–50,000/μL)	9	10 (5–14)	46,000 (36,000–49,000/μL)
Mild (51,000–100,000/μL)	89	10 (2–35)	82,000 (51,000–99,000/μL)

Table 4 delineates the number of acupuncture treatments based on the severity of thrombocytopenia. Of note, nine treatments involved patients with platelet levels <50,000/μL. The most common reason for acupuncture referral was pain (40%), followed by stress/anxiety (28%), and fatigue (18%). Less often, patients wanted to utilize acupuncture to alleviate nausea and vomiting, or peripheral neuropathy (6% each). From a TCM perspective, the most common diagnoses included Qi and Blood Stagnation (26%), Spleen Qi Deficiency (17%), and Liver Qi Stagnation (16%) (Table 5).

**Table 4.** Distribution of Acupuncture Treatments by Platelet Count

*Platelets*	*Visits*
>100,000/μL	2037
<100,000/μL	98
90,000–99,000/μL	37
80,000–89,000/μL	16
70,000–79,000/μL	19
60,000–69,000/μL	11
50,000–59,000/μL	6
40,000–49,000/μL	8
30,000–39,000/μL	1
≤29,000/μL	0

**Table 5.** Chief Complaints and TCM Diagnoses

*Parameters*	# of patients (%)
**Chief complaint**	
Pain	39 (39.8%)
Stress/anxiety	27 (27.6%)
Fatigue	18 (18.4%)
Nausea/vomiting	6 (6.1%)
Peripheral neuropathy	6 (6.1%)
Other	2 (2.0%)
**TCM diagnosis**	
Qi & Blood Stagnation	26 (25.5%)
Spleen Qi Deficiency	17 (16.7%)
Liver Qi Stagnation	16 (15.7%)
Spleen & Kidney Yang Deficiency	8 (7.8%)
Bi Syndrome	6 (5.9%)
Other	29 (28.4%)

TCM, Traditional Chinese Medicine.

Data regarding concurrent use of medications and/or dietary supplements/botanicals that can affect coagulation were also collected. Table 6 provides results regarding medication and dietary supplement/botanical use. Oxycontin was the most frequently encountered medication (27% of visits), followed by dexamethasone (7% of visits). The maximum number of concurrent medications for any single visit was three. Fish oil was the most frequently encountered nutraceutical (48% of visits). Turmeric (curcumin; *Curcuma* spp.), vitamin E, and vitamin K were taken at 34%, 11%, and 11% of visits, respectively. The maximum number of concurrent nutraceuticals/botanicals at a single visit was five. For visits in which patients took both coagulation-affecting medications and nutraceuticals/botanicals, the maximum combined number was five (2 medications, 3 nutraceuticals/botanicals). No adverse events were reported in any of the visits.

**Table 6.** Visits Involving Medications, Nutraceuticals, & Botanicals Associated with Blood Thinning

*Conventional medication*	*# of visits*
Oxycontin^®^	27
Dexamethasone	7
Arixtra^®^	6
Prednisone	5
Ibuprofen	2
Coumadin^®^	2
Percocet^®^	1
Motrin^®^	1
Heparin/Lovenox^®^	1
**Maximum number of medications at a single vi**si**t**	**3**

*Nutraceutical or botanical*	*# of visits*
Fish oil	46
Turmeric (curcumin; *Curcuma* spp.)	34
Vitamin E	11
Vitamin K	11
Ginger *(Zingiber officinale)*	9
Licorice (*Glycyrrhiza* spp.)	4
American ginseng	3
Milk thistle (*Silybum marianum*)	1
Oregon grape (*Mahonia* spp.)	1
Quercetin	1
**Maximum number of nutraceuticals at a single visit**	**5**
**Maximum combination of medications and nutraceuticals/botanicals**	**2 medications+3 nutraceuticals/botanicals**

## Discussion

Several large prospective studies evaluated the safety of acupuncture treatments. In 2001, MacPherson and colleagues reported no serious adverse events that required hospital admission in 34,407 acupuncture treatments.^9^ Minor adverse events occurred at a rate of 1.3 per 1000 treatments (95% confidence interval: 0.9–1.7), which included severe nausea and actual fainting (*n*=12), unexpected, severe and prolonged aggravation of symptoms (*n*=7), and prolonged and unacceptable pain and bruising (n=5).^9^ Patients undergoing cancer treatment are at an increased risk of developing adverse events because of having pancytopenias and compromised immune function, making these patients more susceptible to bruising or bleeding, hematoma, and possible infection.

Wedong et al.^10^ completed a review of acupuncture practices in 2010. They noted that the rate of adverse events can be decreased significantly by adhering to the following requirements: “using a specific type of thin needle, mild manual stimulation administered at a shallow depth, by an experienced acupuncturist at an academic cancer center with an established acupuncture program.” As such, these researchers suggested that oncology patients with an absolute neutrophil count of <500/μL or a platelet count <25,000/μL are not suitable candidates for acupuncture treatment.

The discrepancies between the ASCO guidelines^11^ and those proposed by Wedong's review^10^ highlight the need to create cohesive safety guidelines backed by clinical research, particularly with regard to patients who are at increased risk for adverse events. The current study, despite its small sample size and retrospective nature, demonstrated that acupuncture can be administered safely and successfully to adult oncology patients with moderate thrombocytopenia. In the current study, 9 oncology patients being treated at an integrated medical facility received acupuncture safely with platelet levels below the 50,000/μL guidelines set by ASCO.^11^ These results, in conjunction with the findings of Ladas et al.'s pediatric study^7^ and Wedong et al.'s review,^10^ suggest that the threshold at which patients should be denied acupuncture may be lowered safely. It is imperative to set these guidelines accurately so that patients are granted care that will potentially increase their ability to tolerate the side-effects of conventional cancer therapies while avoiding unnecessary risks. For many patients in the metastatic setting, relief of subjective complaints is a top priority.

It should be noted that, similar to the review by Wedong et al.^10^ and Ladas, et al.^7^ all practitioners who participated in the care of patients included in the current analysis had undergone additional training with regard to safety, acupuncture technique, and management of patients with cancer. Similar to the pediatric retrospective case series,^7^ the acupuncture described in the current report utilized Japanese J-type Seirin needles. The needles were not inserted past ½″ in depth. If manipulation of the needle was required, it was performed gently to obtain the De Qi sensation. The unique design of Seirin needles may facilitate a decreased risk of adverse events.

The inclusion of nutraceuticals/botanicals is unique to this study. Integrative health care facilities have gained favor with the public and momentum within the field of oncology; however, the safety data on this combined model of care is limited. As multiple nutraceuticals/botanicals may affect bleeding, included these were in the current study's data set. To the current authors' knowledge, this is the first study to assess whether nutraceuticals/botanicals, in combination with conventional care, have synergistic impacts on increasing acupuncture's rate of adverse events. The preliminary evidence in this study lays the foundation to support the notion that acupuncture can be used safely with this high-need patient population within an integrative model of care.

## Conclusions

To the best of the current authors' knowledge, this is the first retrospective review to assess the incidence of side-effects of acupuncture in thrombocytopenic adult oncology patients within an integrative medical facility. In this analysis, no adverse events specifically referable to acupuncture (i.e., bleeding, bruising, soreness at the site) were observed.

Limitations of this study include the retrospective nature of the case series. Based on these data, further prospective studies are planned to assess side-effects resulting from the use of acupuncture in adult oncology patients. The lack of adverse events noted in both this study involving adult oncology patients and the prior pediatric study suggests that the ASCO Clinical Practice Guidelines,^11^ which allow acupuncture in patients with platelets of ≥50,000/μL may be further reduced to the levels proposed by Wedong et al.^10^ Prospective studies are needed to confirm that this guideline can be lowered safely to permit greater access to acupuncture therapy.
